# Scaling up family medicine training in Gezira, Sudan – a 2-year in-service master programme using modern information and communication technology: a survey study

**DOI:** 10.1186/1478-4491-12-3

**Published:** 2014-01-21

**Authors:** Khalid G Mohamed, Steinar Hunskaar, Samira Hamid Abdelrahman, Elfatih M Malik

**Affiliations:** 1 Department of Family and Community Medicine, University of Gezira, Medani, Sudan; 2 Department of Global Public Health and Primary Care, University of Bergen, P.O. Box 7804, NO-5020 Bergen, Norway; 3 Ministry of Health, Medani, Gezira State, Sudan

**Keywords:** Family medicine, In-service training, Telemedicine, Sudan, Curriculum development

## Abstract

**Background:**

In 2010 the Gezira Family Medicine Project (GFMP) was initiated in Gezira state, Sudan, designed as an in-service training model. The project is a collaboration project between the University of Gezira, which aims to provide a 2-year master’s programme in family medicine for practicing doctors, and the Ministry of Health, which facilitates service provision and funds the training programme. This paper presents the programme, the teaching environment, and the first batch of candidates enrolled.

**Methods:**

In this study a self-administered questionnaire was used to collect baseline data at the start of the project from doctors who joined the programme. A checklist was also used to assess the health centres where they work. A total of 188 out of 207 doctors responded (91%), while data were gathered from all 158 health centres (100%) staffed by the programme candidates.

**Results:**

The Gezira model of in-service family medicine training has succeeded in recruiting 207 candidates in its first batch, providing health services in 158 centres, of which 84 had never been served by a doctor before. The curriculum is community oriented. The mean age of doctors was 32.5 years, 57% were males, and 32% were graduates from the University of Gezira. Respondents stated high confidence in practicing some skills such as asthma management and post-abortion uterine evacuation. They were least confident in other skills such as managing depression or inserting an intrauterine device. The majority of health centres was poorly equipped for management of noncommunicable diseases, as only 10% had an electrocardiography machine (ECG), 5% had spirometer, and 1% had a defibrillator.

**Conclusions:**

The Gezira model has responded to local health system needs. Use of modern information and communication technology is used to facilitate both health service provision and training. The GFMP represents an example of a large-volume scaling-up programme of family medicine in Africa.

## Background

There is a global emphasis on the importance of family medicine and its role as a cornerstone in the modern health system [1]. The International Conference on Primary Health Care (in Alma-Ata, former USSR, 1978) called for urgent and effective national and international action to develop and implement primary health care throughout the world and particularly in developing countries [2]. This was further emphasized and detailed by the 2008 World Health Organization report *Primary Health Care: Now More than Ever*[3].

Many studies have shown that better health service outcomes are related to the quality of primary health care available [3,4]. European countries that have the strongest primary health-care systems could achieve this mainly due to reforms aimed at transferring power and tasks to general practitioners [1,5]. Vertical programmes for diseases such as HIV, malaria, and tuberculosis, and also for vaccinations, should be implemented in the context of primary health-care teams to be more effective and comprehensive [6]. Although much money has been invested in vertical programmes, the overall performance of disease control programmes is generally poor [7].

Family medicine is a very recent discipline worldwide, started in the 1960s as a postgraduate training programme [8]. There are a variety of tasks, roles, and settings for family physicians worldwide [1]. Internationally there are two basic models for family medicine training, hospital-based training and primary care-based training. The latter is usually called primary care in-field-training or in-service training. Many countries use mixed models. Norway and the United Kingdom are examples of countries using mainly in-service training programmes for family physicians. In Norway the specialty period is 5 years, where 4 years should be training at a health centre (in-field) and 1 year at a hospital [9].

Africa experiences 24% of the global burden of disease, but has only 2% of the global supply of doctors [10]. Until recently, the concept of family medicine has not been clearly defined in the context of sub-Saharan Africa [11]. How family physicians can best be used in primary health care in Africa is therefore a pertinent question [12]. The 2nd African Regional WONCA Conference (World Organization of National Colleges, Academies and Academic Associations of General Practitioners/Family Physicians, in Rustenberg, South Africa, 2009) developed a consensus statement on family medicine within an African context, as well as the role and training requirements of family physicians [13]. A number of family medicine training programmes have been established in Africa during the last decade [14]. Implementation of family medicine in Africa faces many challenges; for example, poverty, limited funding, focusing on hospital care, poor training capacity, and drainage of doctors towards rich or more developed countries [10].

Barbara Starfield has claimed that the most successful health systems should plan for between 1000 and 1500 inhabitants per full-time family doctor [15]. The Gezira state of Sudan had 115 medical officers (graduated doctors without postgraduate training) and no family medicine specialist at the start of 2010, reflecting a ratio of primary health-care doctors to population of about 1:32 000. To bridge this huge gap, the Gezira Family Medicine Project (GFMP) was planned. The project aims to train doctors in family medicine by means of a 2-year in-service master’s degree programme.

This paper describes the GFMP, including its implementation, the different roles of partners, the curriculum, and the use of modern information and communication technology. The paper also focuses on the background and baseline competences of the recruited candidates, characteristics of primary health centres used for the training, and instruments and methods used in the training.

## Methods

The GFMP is the first of its kind in the region. A scientific evaluation of the project was therefore planned to be performed alongside with the first batch of candidates. The evaluation is planned to cover the development of the organization (health centres, equipment), the students (fulfilment of the programme, competence in family medicine and practical skills, patient-centred approach), teaching methods (use of distance learning and telemedicine), and some patient perspectives (catchment area, doctor’s knowledge of the patient, management of major conditions).

### Study area

Sudan lies in north-east sub-Saharan Africa covering 1 861 484 km^2^, with an estimated population of 34 million (in 2012). According to official reports from the Ministry of Health, Sudan has an infant mortality rate of 68 deaths per 1000 live births, maternal mortality of 750 deaths/100 000 live births, and a life expectancy at birth of 55 years. Gezira state is one of 17 states in Sudan, centrally located in the country, with a total area of about 25 500 km^2^. Gezira state has a total population of about 3.7 million. Rural population constitutes about 80% of the total population in Gezira, scattered over more than 3000 villages. According to the state Ministry of Health, the main causes of morbidity and mortality are infectious and parasitic diseases. Noncommunicable diseases are also emerging due to the change in socioeconomic and lifestyle conditions.

### Study design

A survey study was designed to collect baseline data from both the GFMP candidates (medical doctors) and from the health centres (training sites). The same design will be used to collect data after graduation, thus defining a follow-up study of the first student batch (cohort observational design).

### Study population

The study population comprises the GFMP candidates and the health centres selected for the project. A total of 188 (91%) candidates responded and filled in the questionnaire. All health centres (158) were reviewed in the study.

### Data collection

Data were collected using a checklist and a self-administered questionnaire. The list and the questionnaire were both developed by the GFMP project group and discussed by senior researchers in the field. The checklist was used to collect data from the health centres involved in the project. The data were collected by both doctors and by the administrative staff from GFMP headquarters. Examples of variables included are location, distance to the nearest main hospital and health centre, buildings/rooms, available laboratory services, vaccination programme, available medications, diagnostic and therapeutic equipment, staff, opening hours, and workload.

All doctors joining the project filled in a comprehensive anonymous questionnaire about themselves, their background, and their previous experience. The questionnaire also included information about their style of practice, their participation in maternal and childhood care, their competence as leaders for the health team, and their role in all health services: prevention, treatment, patient education, health promotion, and rehabilitation. Moreover, the questionnaire recorded their workload, and their interest in family medicine as a specialty. Their self-assessed confidence and competence in performing 46 different procedures was reported using a five-level score (from not able to very confident). The doctors’ questionnaire also included the Patient–Practitioner Orientation Scale, which is an 18-item scale on patient-centredness [16]. Data from the Patient–Practitioner Orientation Scale are not presented in this paper. The questionnaire data were collected after each student had started the programme, and for the last questionnaires this meant several weeks into the first term.

### Ethical and privacy approvals and data analyses

The study was reviewed and approved by the Ethical review committee at the Ministry of Health, Gezira State, Sudan. The study was also reviewed by the Regional Committee for Medical and Health Research Ethics, Western Norway. Privacy issues and patients’ file management related to the scientific evaluation were also approved by the Norwegian Data Protection Official for Research.

Data management and statistical analyses were done by the IBM SPSS® program version 19 (supplier: IBM corp., Armonk, NY, USA). Results are presented as descriptive statistics with means and proportions and percentages.

## Results

### Gezira Family Medicine Project (GFMP)

In early 2010, following advocacy campaigns about the GFMP, a total of 207 doctors were accepted for the programme, distributed to 158 health centres. The project is a product of collaboration between several partners including the Ministry of Health at Gezira state, University of Gezira, the Sudan Medical Specialization Board, the National Health Insurance Fund, and the community. The mission of the project is to provide high-quality, accessible, and affordable primary care-based health services.

The roles of the different partners were specified from the start. The Faculty of Medicine, University of Gezira, was responsible for the academic training component including curriculum development. The Ministry of Health was responsible for providing the health centres with the required staff, including doctors, equipment, and buildings, in addition to paying the candidates’ tuition fees. After the recruitment of doctors, the health centres were analysed according to the need for expanding the workforce and, when needed, other health-care workers such as laboratory technicians and nurses were employed and paid by the Ministry. The Sudan Medical Specialization Board approved that any successful graduate from the 2-year master’s programme eventually can continue for 2 years more in order to obtain the full MD degree in family medicine at the national level. The local communities in Gezira state provided housing for the doctors, especially in rural areas. The National Health Insurance Fund has decided to provide health insurance services at all health centres included in the GFMP.

Results and descriptions here will cover the three main components of the GFMP; training, service, and information and communication technology.

#### The training component

The University of Gezira adopted a community-oriented training approach. The Faculty of Medicine prepared an academic plan for the GFMP through curriculum workshops, led by family physicians together with experts from other medical disciplines. The main feature of the approved curriculum was the in-service approach. This implies that the curriculum was designed and structured to meet both training and service requirements. The awarded degree was decided to be a Master of Science in Family Medicine.

### Structure of the programme

The structure of the 2-year programme is based on four semesters. The first three semesters, with a total academic load of 48 credit-hours (one credit-hour equals 15 theoretical-hours or 30 practical/clinical-hours) are divided into teaching blocks (modules). The fourth semester has been allocated to research (master’s dissertation). The distribution of credit-hours between the specialties is described in Table 1. Modern pedagogic principles are used as demonstrated in Table 2, which shows the training methods used in the GFMP.

**Table 1:** Summary of courses in the Gezira Family Medicine Project curriculum

**Course name**	**Credit-hours**	**%**
Family medicine	8	16.6
Community medicine	3	6.3
Internal medicine	5	10.4
Diagnostic imaging and laboratory medicine	2	4.1
Research methodology	1	2.0
Paediatrics and child health	5	10.4
Obstetrics and gynaecology	5	10.4
Otolaryngology	3	6.3
Surgery and orthopaedics	4	8.3
Psychiatry	3	6.3
Dermatology	3	6.3
Ophthalmology	3	6.3
Accident and emergency medicine	3	6.3
Total	48	100

**Table 2:** Training methods used in the Gezira Family Medicine Project

**Training activity**	**Way of conducting**	**Time of the training**	**Aim of the training**
Introductory course	Meeting at the university	At the start of the master’s course	To cover important areas in family and community medicine, before start
Distance learning	Formal Internet-based lectures, tutorials or discussions, using Web Ex program (virtual classroom); lecturer communicates with candidates (voice and picture), share desktop, slides, films, and documents	Usually at the end of the day, evenings, or weekends	To teach the different disciplines in rotations (medicine paediatrics, and so forth)
Hospital visits	Clinical rounds, outpatient clinic, referral clinic, theatre, etc.	Once a week	To learn the required clinical skills, candidates should fill their logbooks where all the required skills are listed
Telemedicine	Specialists from all specialties are connected with the candidates by videoconference to discuss real-life cases	One hour during the working day	Both clinical management and case discussion learning
Field supervision	Supervisors visit the candidates at their health centres	During the working day	To assess the setting, attend and evaluate consultations, evaluate the use of the filing system
Primary care work	Candidates are practicing family medicine at their centres	4 days a week	Learning through practice, candidates communicate with specialists and colleagues through videoconferencing
Courses	Crash courses arranged at the university	Usually 3 to 5 days per course	To cover certain important topics (examples: mental health, malaria and HIV)
Monthly meetings	Meeting at the university	Once a month	Family medicine teaching and administrative issues
Electronic library	Every candidate is equipped with a laptop computer and free wireless Internet	At any time	Evidence-based medicine: candidates should know national and international resources, guidelines, medical websites, etc.
Classical library	Available at the university, some books were distributed free to the candidates	During the university days	Mainly for background knowledge

The candidates were assessed through a series of examinations at the end of each clinical module. The electronic medical records were used to assess their real practice, which includes topics such as clinical governance, holistic approach, evidence-based practice, and guideline follow up. This assessment was done by checking a random number of patients’ records. The assessor discussed the findings, notes, decisions, management, and so forth, with the student, and gave feedback in a direct and personal audit process.

The candidate’s social accountability, community mobilization, and health-promotion activities were also evaluated. The candidates were asked to document their activities in the community through pictures, reports, and minutes of relevant meetings. At the examination the students were asked to present the data (which were collected by the community members) regarding the population, age, and sex register and any chronic diseases’ registries in the catchment area/practice population. The students also displayed pictures and reports about any community participation regarding rehabilitation of the health centre. Any participation of the doctor in health promotion in the community or at schools should be presented and documented. All candidates had to present and discuss a Google map poster of his/her catchment area, and to present the population pyramid of the village or catchment area.

### Enrollment of candidates

All applicants were interviewed before their intake. There was no restriction according to age or medical experience except for performed internship (12 months) after graduation and permanent registration in Sudan Medical Council.

#### The service presentation component

The GFMP is also a matter of health system reform, first by transforming the health system from its verticality to a more horizontal system, focusing on the health team rather than the health programmes, and secondly by providing more resources to primary health care regarding staff, equipment, and buildings. The expected role of the family medicine candidates is to provide continuous and comprehensive care for all people of all ages and both genders with their broad spectrum of needs, health problems, and diseases [3]. They are supposed to mobilize the community resources and to respect its needs. They are also supposed to lead the health team within the catchment area.

#### Information and communication technology component

These technologies are used to facilitate both training and service. They include telemedicine using the videoconferencing software program ooVoo® [17]. Family doctors were connected with hospital specialists from different medical specialties, and clinical signs or investigations such as electrocardiography (ECG) could be shown and discussed. For distance learning, the software Cisco WebEx® [18] is used. Candidates were connected electronically to a virtual classroom with full interaction of life voice, picture, and chatting. An electronic filing system was developed for the GFMP (Family Clinic®). This system incorporates also the ICD-10 diagnostic system, and statistical reports can be generated.

### Health centres – the clinical training environment

The 158 health centres were distributed all over Gezira state, but the majority was clearly rural. The mean distance to the nearest of three main hospitals was 31 km (SD 26). Distribution of the distances was otherwise as follows: less than 10 km (30% of the centres), and more than 50 km (20%). The longest distance was 100 km.

All centres had a consultation room, but otherwise they were very diverse in number of rooms (Table 3). A few centres had an available X-ray and/or ultrasound room and major operation room because of sharing facilities with small local village public hospitals. Availability of laboratory equipment, instruments, and some other diagnostic or therapeutic equipment is shown in Table 4. Most centres had the most relevant blood tests available, while more advanced equipment was rather seldom present. Typical opening hours for a health centre are 7 to 8 hours 5 days a week, with rather small variations (data not shown).

**Table 3:** **Rooms available at the health centres of the Gezira Family Medicine Project at baseline (*N *** **= 158)**

**Description of room**	***n***	**%**
Doctor’s consultation room	158	100
Laboratory room	149	94
Pharmacy	136	86
Ward/observation beds	136	86
Vaccination room	102	65
Minor surgery room	86	54
Maternal room	70	44
Storage room	57	36
Registration and filing room	50	32
Nutrition room	30	19
X-ray or ultrasound room	25	16
Major surgery room	21	13

**Table 4:** **Laboratory equipment, instruments, and other diagnostic or therapeutic equipment available at the health centres of the Gezira Family Medicine Project at baseline (*N *** **= 158)**

**Description**	***n***	**%**
Laboratory investigations		
Blood film for malaria	142	90
Pregnancy test	137	87
Standard urinalysis	136	86
Standard stool investigation	134	85
Erythrocyte sedimentation rate	134	85
Haemoglobin	132	84
Total white blood cell count	132	84
Widal test for typhoid	121	77
Blood glucose	111	70
Immunochromatographic test for malaria	57	36
HIV test	25	16
Diagnostic or therapeutic equipment		
Microscope	140	89
Sphygmomanometer	131	83
Centrifuge	124	78
Colorimeter	111	69
Nebulizer	108	68
Surgical equipment for minor surgery	92	58
Autoclave	79	50
Gynaecological examination equipment	43	27
Electrocardiography machine (ECG)	16	10
Ear syringe	16	10
Surgical equipment for major surgery	14	9
Ophthalmoscope	8	5
Spirometer	7	4
Emergency bag with relevant content	7	4
Defibrillator	1	1

### The students – background and baseline experience and skills

**Figure 1:**
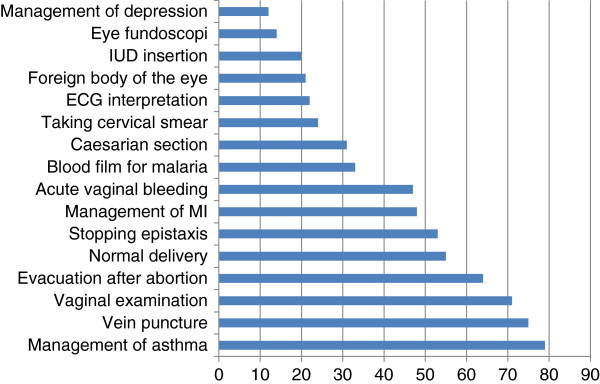
**Self-assessed evaluation of the ability to perform 16 of a total of 46 recorded skills.** Data shown are the sum of the scores (%) from the alternatives 'very confident’ and 'confident’ (percentages) on a category scale with five alternatives ('very confident’, 'confident’, 'not fully confident’, 'uncertain’, and 'not able’). The data were collected after each student had started the programme, and for the last questionnaires this meant several weeks into the first term. ECG, electrocardiography; IUD, intrauterine device; MI, myocardial infarction.

The students’ mean age was 32.5 years; the male candidates were somewhat older than the women (Table 5). Males also had a wider age range, and more working experience as 12% of them had more than 10 years of experience before entering the programme. About one third of the students were graduates of the University of Gezira, while 6% came from outside Sudan. A variety of reasons for choosing the family medicine specialty was given (Table 5). Examples of self-assessed evaluations of the ability to perform 46 different skills are shown in Figure 1. Almost 79% were confident or very confident in managing asthma, some confidence was also found for some major surgical operations, while only 20% felt confident in intrauterine device insertion and 12% in managing depression.

**Table 5:** **Description of the students at the start of the Gezira Family Medicine Project at baseline (*N *** **= 207)**

**Description**	**Males (*n *** **= 118)**	**Females (*n *** **= 89)**	**All (*N *** **= 207)**
Gender	57	43	100
Age (years)			
Mean (standard deviation)	34.0 (7.9)	30.4 (4.5)	32.5 (6.9)
Median	32	30	30
25th to 75th quartiles	28 to 36	27 to 33	28 to 35
Range	24 to 62	24 to 47	24 to 62
University background			
Gezira	27	38	32
Khartoum	12	8	10
Other Sudanese	48	50	49
Other countries	9	3	6
Missing	4	1	3
Working experience (years)			
< 2	30	44	37
2 to 5	41	39	39
6 to 10	17	15	16
>10	12	2	8
Reason(s) for choosing family medicine			
Economy	29	18	24
Easy specialty	13	4	9
Short duration of programme	20	23	21
Only available	31	31	31
Other reasons	21	24	22

Results presented as percentages unless otherwise stated.

## Discussion

The GFMP succeeded in scaling up family medicine training in Sudan by recruiting 207 candidates who were providing health services in 158 health centres, 84 of which (53%) had never been served by a doctor before the project.

Recruitment of doctors to join family medicine training programmes in developing countries has been a major challenge [19,20]. This was not the case in the GFMP. One third of the candidates were graduated from the University of Gezira followed by one tenth from the nearby University of Khartoum. This reflects the role of the local universities in developing its surrounding communities by being the main provider of health-care personnel for the area.

Distance to the nearest main hospital reflects the rural nature of Gezira state, including widespread population, inadequate insurance coverage, and limited accessibility to health services. However, in the rainy season transportation of patients over even rather short distances may be hindered by flooding canals and destroyed roads.

The available diagnostic or therapeutic equipment reflects the practice in these centres before the GFMP. Equipment necessary for noncommunicable diseases and emergency cases were inadequate. This is in spite of evidence showing these problems as emerging threats while the health system shows clear gaps in low-income and middle-income countries [21]. Until now Sudan has had an almost absence of a well-functioning referral system. Combined with low medical competence and a lack of even basic equipment, this makes it understandable that many primary care patients are treated at a specialist level. Only 16% had HIV tests available. Such patients are usually referred to specific HIV centres or to secondary care even before confirming the diagnosis. This weak contact between HIV patients and primary care services represent one of the cons of vertical disease programmes [7], seen also in Sudan.

The candidates stated clearly a lack of confidence in the management of depression. This may be due to the scarcity of psychiatric illness presented at a primary care level, but might also be due to insufficient attention devoted to mental health issues in general [22]. The candidates stated also high confidence in caesarean section, but much less confidence in inserting an intrauterine device, reflecting the need for family planning skills [23]. Although malaria is a common disease we found that only about one third of the candidates stated that they were confident in detecting malaria by means of a blood film.

Many developing countries are now aware of the importance of family medicine and its commitment to the Millennium Development Goals [24]. Research from regions such as Europe reflects evidence that family medicine plays an important role in the development of the health system and in improving primary health-care services outcomes [5]. Unfortunately there is still scarce research providing such evidence in sub-Saharan Africa.

Comprehensive studies regarding primary care doctors in Sudan are few, but some studies describe clinical skills in certain areas. A study on malaria showed that the rate of false-positive diagnosis of malaria by clinicians was 76% [25], emphasizing the importance of training for primary care doctors. Another study showed good outcome after a 3-week programme for doctors working at rural hospitals in Sudan [26]; post-training field assessment revealed that 60 to 80% of the trainees were using recently learned techniques.

A study from Saudi Arabia described a 1-year diploma programme in family medicine that started in 2007 [27]. The sample size was 34 trainees (all of the candidates in the second batch), and the response rate was 91%. The short training period (1 year) was reported as a barrier by 84% of the trainees. But the importance of increased use of information and communication technology in order to improve the training was highly emphasized by the candidates in the study. The GFMP could solve two of the problems recognized, as the duration of training is longer and the use of information and communication technology is much more comprehensive.

A study from Brazil and Chile evaluated an in-service model of training for primary health-care workers. Based on pretests, post-tests, and evaluations of students’ projects, it was demonstrated that participants had increased knowledge as well as skills after in-service training [28]. Another study from Brazil evaluated the impact of a family medicine-based programme on the infant mortality rate during 1990 to 2002 [29]. Such studies emphasize that new educational developments such as the GFMP should be followed by evaluation research and, if possible, clinical outcomes. We have developed a research plan to investigate a variety of effects of the GFMP.

The consensus document from the Annual PRIMAFAMED Network Conference held in Victoria Falls, Zimbabwe, in 2012 stated the GFMP as a good example for scaling up family medicine and primary health care in Africa [30]. This has been important feedback from the scientific community, showing that we are on the right track and that our ideas and models are appreciated. The GFMP is thus now a well-known example that inspires other countries in the region. This fact is also inducing a commitment to conduct a scientific evaluation of the programme.

### Strengths and limitations of the study

Our cross-sectional study included all trainees and health centres in the GFMP, resulting in a representative and comprehensive information. The response rate of the doctors’ questionnaire was 91%, with only 19 candidates not responding. The response rate for health centres’ questionnaire was 100%, giving an important and complete database about the setting for training and service prior to the programme launch. Most parts of the questionnaires had face and content validity as they recorded simple and factual data that were checked by both administrative and research staff of the project.

One clear study limitation is the self-reporting of clinical skills and other self-evaluations by the trainees. Validity and reliability studies could have increased the quality of such data. However, self-report represents a feasible method of data collection, since it requires little administration and allows large samples to be included [31]. Objective checking of the competence of 207 candidates regarding how they perform 46 different clinical skills is not obtainable in a running training enterprise.

## Conclusions

The GFMP represents a modern model in family medicine training that responds to local needs. The project copes with the regional strategy for up-scaling of family medicine training. Information and communication technology is a cornerstone in performing such a mission. The GFMP will hopefully provide a suitable model to other low-income and middle-income countries that share similar health-care challenges as Gezira state in Sudan.

## Abbreviations

GFMP: Gezira Family Medicine Project.

## Competing interests

KGM is an assistant professor at the University of Gezira, Sudan, and the director of the GFMP at the Ministry of Health. SH is a professor of family medicine at the University of Bergen, Norway, is honorary professor at the University of Gezira, and acts as a consultant for the GFMP and Ministry of Health in Gezira state. EMM is a researcher and, at the present time (2013), is the state Minister of Health in Gezira state, Sudan. SHA is a professor of community medicine at the University of Gezira, and is also the Dean of the Blue Nile National Institute for Communicable Diseases.

## Authors’ contributions

All authors contributed to the design and conception of this study. All authors revised the manuscript critically and gave final approval for the manuscript to be published. KGM and SH contributed to data collection, interpretation and analysis. KGM drafted the first draft while the other authors revised it critically and gave ideas. All authors read and approved the final manuscript.
